# Preliminary Evaluation of the Clinical Benefit of a Novel Visual Rehabilitation Program in Patients Implanted with Trifocal Diffractive Intraocular Lenses: A Blinded Randomized Placebo-Controlled Clinical Trial

**DOI:** 10.3390/brainsci11091181

**Published:** 2021-09-08

**Authors:** David P. Piñero, Ainhoa Molina-Martin, María L. Ramón, José L. Rincón, Cristian Fernández, Dolores de Fez, Juan F. Arenillas, Luis Leal-Vega, María Begoña Coco-Martín, Miguel J. Maldonado

**Affiliations:** 1 Group of Optics and Visual Perception, Department of Optics, Pharmacology and Anatomy, University of Alicante, 03690 Alicante, Spain; ainhoa.molina@ua.es (A.M.-M.); dolores.fez@ua.es (D.d.F.); 2 Department of Ophthalmology, Vithas Medimar International Hospital, 03016 Alicante, Spain; mramoncano@gmail.com (M.L.R.); jlrincon40@hotmail.com (J.L.R.); cristianfermar@gmail.com (C.F.); 3 Group of Applied Clinical Neurosciences and Advanced Data Analysis, Neurology Department, Faculty of Medicine, University of Valladolid, 47005 Valladolid, Spain; juanfarenillas@gmail.com (J.F.A.); luis.leal.vega.1213@gmail.com (L.L.-V.); 4 Stroke Unit and Stroke Program, Department of Neurology, University Clinical Hospital, University of Valladolid, 47003 Valladolid, Spain; 5 Grupo de Cirugía Refractiva y Rehabilitación Visual, Instituto Universitario de Oftalmobiología Aplicada (IOBA), Universidad de Valladolid, 47011 Valladolid, Spain; maldonado@ioba.med.uva.es

**Keywords:** multifocal IOL, contrast sensitivity, visual rehabilitation, Gabor patches, trifocal diffractive IOL, visual training

## Abstract

The authors of this study evaluated the potential benefit on visual performance of a novel 3 week visual rehabilitation program based on the use of Gabor patches in patients undergoing bilateral cataract surgery with the implantation of two models of trifocal diffractive intraocular lens (IOL). A total of 30 patients were randomly assigned to two groups: a study group (15 patients) that used a videogame based on Gabor patches and a placebo group (15 patients) that used a videogame without specific stimuli for improving visual performance. No statistically significant differences between groups were found in distance, intermediate, and near post-training visual acuity (*p* ≥ 0.15). Significantly better distance contrast sensitivity (CS) was found for the spatial frequencies of 6 (*p* = 0.02) and 12 cpd (*p* = 0.01) in the study group. Likewise, significantly better values of near CS were found in the study group compared to the placebo group for the spatial frequency of 1.5 cpd (*p* = 0.02). In conclusion, a 3 week visual rehabilitation program based on the use of Gabor patches in the immediate postoperative period after the bilateral implantation of trifocal diffractive IOLs seems to be beneficial for improving both distance and near visual performance achieved with the implant.

## 1. Introduction

The implantation of multifocal intraocular lenses (IOLs) after cataract surgery has become a common routine in many ophthalmological centers because this type of implant allows for successful visual restoration at all distances [1]. Indeed, multifocal IOLs are superior to conventional monofocal IOLs in terms of spectacle independence [1]. Among multifocal implants, trifocal diffractive IOLs have been shown to provide a wide range of focus, with good levels of distance, intermediate visual acuity, and near visual acuity, thus making them one of the most effective options for presbyopia correction [2,3,4]. However, multifocal IOLs are associated with a higher prevalence of visual disturbances, including suboptimal visual acuity, decreased contrast sensitivity [5,6], and haloes or glare [7], thus leading to patient dissatisfaction [8] and in the most severe cases to IOL explantation [9].

Several modifiable factors can lead to visual disturbances after cataract surgery with multifocal IOL implantation, such as tear film instability, IOL decentration, and the generation of some level of posterior capsular opacification [10,11]. All these conditions can be solved or treated. However, the problems of visual quality and patient satisfaction are sometimes related to neuroadaptation [12], with the activation of certain brain areas playing a role in adaptation to multifocal IOLs [13,14]. Previous research has shown an association of the activation of cortical areas dedicated to attention (frontoparietal circuits), learning and cognitive control (cingulate), and task goals (caudate) with the visual difficulties reported by patients implanted with multifocal IOLs [13]. For this reason, it has been suggested that the improvement of visual attention and procedural learning networks may be an essential part of the initial process of neuroadaptation to multifocal IOLs, promoting improvements in visual acuity, contrast sensitivity, and patient satisfaction independently of optical factors [14]. In this context, the development of training programs facilitating these neural changes that lead to neuroadaptation has been proposed as a potentially useful option to avoid patient dissatisfaction after multifocal IOL implantation [12]. 

Visual training using videogames and specific types of stimuli have been shown to be effective for improving visual function in different conditions [12,15,16,17,18], even in adult patients [19,20,21,22,23,24,25]. Specifically, the use of sinusoidal gratings with a neutral background can cause selective cortical responses for orientation and contrast [26]. Likewise, gaming can promote brain adaptations and improve functional connectivity, with significant beneficial effects in a great variety of brain functions, including attention, cognitive control, and visuospatial skills [27,28]. Therefore, a combination of videogames with the stimulation of sinusoidal gratings seems to be a potentially effective tool for improving visual function in adult patients [12]. Kaymak et al. [15] reported the use of this type of visual training in patients implanted with multifocal IOLs for the first time, performing a computer-based visual training in only one eye and serving the untrained fellow eye as a control. However, the visual training program was not gamified and the used sinusoidal gratings did not have a smooth transition in their edges (as recommended to evaluate and stimulate a specific channel [29]) and stimuli to promote the activation of cortical areas related to neuroadaptation to multifocality [13]. Recently, a new computerized game was developed to train the visual function of patients implanted with multifocal IOLs while considering all of these aspects in an attempt to optimizing the visual response to multifocality. In the current clinical trial, the efficacy of this new training program was investigated and compared with that of a placebo software that did not have the following peculiarities to promote neuroadaptation: use of Gabor patches that are Gaussian-windowed sinewave gratings, a game environment and activities and exercises promoting the activation of the cortical areas related to attention, learning, and cognitive control; and task goals with the difficulties reported [13]. Specifically, the aim of the current clinical trial was to evaluate the real clinical benefit on visual performance of a novel visual training program based on the use of Gabor patches in patients undergoing bilateral cataract surgery with the implantation of a trifocal diffractive IOL.

## 2. Materials and Methods

### 2.1. Patients

This blinded randomized placebo-controlled clinical trial included 30 subjects who underwent uneventful bilateral cataract surgery with the implantation of trifocal diffractive IOLs at the Department of Ophthalmology of the Vithas Medimar International Hospital (Alicante, Spain) between September 2020 and May 2021 and were willing to perform a visual training program in the immediate postoperative period. Subjects were randomly assigned to use the visual training program after surgery or a placebo program using a random number sequence. Written informed consent was obtained from all participants before their enrollment in the study following the tenets of the Declaration of Helsinki. This clinical trial protocol was approved by the ethics committee of the University of Alicante (UA-2019-02-20), as well as by the Medical Ethics Committee of the University Clinic Hospital of Valladolid (CASVE-NM-20-437). This clinical trial was also registered in https://clinicaltrials.gov/(NCT04985097) (Accessed 8 September 2021).

The following inclusion criteria were established for the study:

(a) Patients who had undergone refractive lens exchange surgery for the correction of presbyopia at least 1 week before the evaluation visit.
(b) Patients implanted with trifocal diffractive IOLs.
(c) Availability and motivation to perform the assigned visual training.
(d) Availability to attend all follow-up visits.

Exclusion criteria included:

(a) Patients implanted with monofocal, extended depth of focus (EDOF), or refractive multifocal IOLs.
(b) Intraoperative complications leading to significant visual sequelae.
(c) Neurological disorders.
(d) Any active ocular disease.
(e) Other previous ocular surgeries, including laser corneal refractive surgery.
(f) Irregular cornea.
(g) Illiteracy.
(h) Any type of psychological disorder.

### 2.2. Surgical Intervention

All surgical interventions were performed by three experienced ophthalmologists (M.L.R., J.L.R. and C.F.) under topical anesthesia. The self-sealing incision, injection of ophthalmic viscosurgical device (OVD), capsulorrhexis, phacoemulsification, irrigation/aspiration of cortical material, and IOL implantation in the capsular bag were performed as standard procedures. IOL power calculations were performed using the SRK-T formula. A total of two different types of trifocal diffractive IOL were implanted depending on clinical characteristics and surgeon preference: Finevision POD F (PhysIOL, Liège, Belgium) and RayOne (Rayner Intraocular Lenses, Ltd., Worthing, UK). Accordingly, two subgroups of patients were created in both the placebo and study groups: patients implanted with the Finevision trifocal diffractive IOL (placebo 6 vs. study 7) and those implanted with the RayOne IOL (placebo 9 vs. study 8).

Subjects were asked to participate in the clinical trial after the intervention and were included as participants within seven postoperative days.

### 2.3. Clinical Protocol

#### 2.3.1. Clinical Examination

The baseline examination, which was performed after surgery and before initiating the visual training, consisted of a complete visual examination including corneal topography, pupillometry, slit lamp biomicroscopy, manifest refraction, measurement of monocular and binocular uncorrected (UDVA) and corrected distance visual acuity (CDVA), measurement of monocular and binocular uncorrected (UIVA) and distance-corrected intermediate (DCIVA) visual acuity measured at 1 m, measurement of monocular and binocular uncorrected (UNVA) and distance-corrected near visual acuity (DCNVA) measured at 40 cm, measurement of monocular and binocular mesopic distance (4 m) contrast sensitivity (CSV-1000E, VectorVision, Greenville, SC, USA), near mesopic contrast sensitivity measured with the Optictrain-CS (Proconsi SL, León, Spain) and Optopad-CSF systems (University of Alicante and Valencia, Alicante and Valencia, Spain) at 40 cm, measurement of monocular and binocular near photopic contrast sensitivity with the Pelli–Robson test at 40 cm, and measurement of the binocular defocus curve considering a range of vergence demands from +1.00 to −3.50 D. This same examination was also performed after finishing the visual training program. All these clinical measures, except those obtained with the Optopad-CSF and Optictrain-CS tests, were performed by the same experienced optometrist (D.P.L.), who was unaware of the specific group in which the subject had been included. 

Near contrast sensitivity measurements with the Optopad-CSF and Optictrain-CS tests were conducted by another experienced examiner (A.M.M.) at an observation distance of 40 cm, with the device used for the presentation of stimuli being plugged into the electric power supply and its screen set to the maximum brightness. All measurements were conducted in a dark room after an adaptation period of 5 min. Results were measured in contrast threshold values and were transformed to logCS for statistical purposes. 

The Optopad test allowed for the measurement of the chromatic discrimination thresholds (Optopad-Color) and the contrast sensitivity function (Optopad-CSF) of a visual system by using an iPad as a display screen. To ensure the device’s correct reproduction of the spatial and colorimetric characteristics of the designed stimuli, they were colorimetrically characterized using the 3DLUT method [30]. Specifically, for the measurement of the achromatic CSF, the Optopad-CSF test allowed for the detection of contrast thresholds for the spatial frequencies of 1.5, 3, 6, 12, and 24 cpd using 5 different plates. Each plate contained a decreasing contrast series of sinusoidal gratings in 2 degree circular windows and was arranged in a 4 × 4 grid against an achromatic background with the device’s maximum generable luminance. The patient’s task was to indicate the direction of the sinusoidal grating in the different rows of each plate. On the other hand, the Optictrain-CS contrast sensitivity test was based on the consecutive presentation of Gabor sinusoidal grating stimuli in a 5 degree circular window with the aim of detecting the contrast threshold for the spatial frequencies of 0.5, 1.0, 1.5, 3.0, 4.5, and 6.0 cpd. A portable computer (Vostro15, Dell) was used for the presentation of the stimuli, with a screen that was also colorimetrically characterized using the Gain-offset-gamma method [31]. The patient’s task was to indicate the direction of the Gabor stimulus in a forced choice task to assess whether the contrast was detected. Changes in the contrast of the stimuli were generated following the rules of the Best PEST psychophysical method [32].

#### 2.3.2. Visual Training Programs

Subjects were implanted with trifocal IOLs to free them of spectacles after the intervention, so the visual acuity achieved at distance and near was assumed to be acceptable enough to satisfy the subjects’ needs. For this reason, the training was planned to be performed without any optical correction. Indeed, no patient referred to the need to use glasses to see properly during the period of visual training after surgery.

##### Study Software

**Figure 1:**
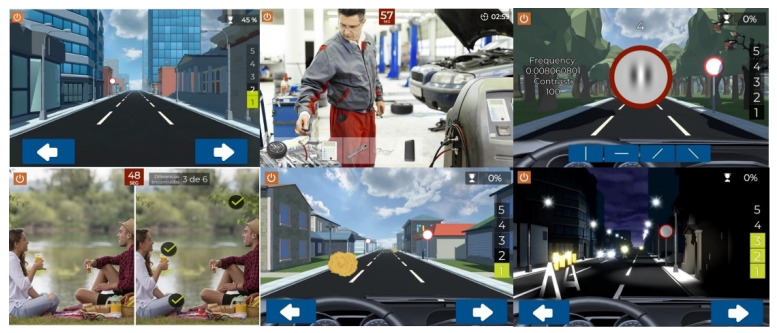
Summary of different screens used during training with the Optictrain software.

Patients that participated in the study were randomly assigned to one of two groups: 15 patients were included in the study group and performed visual training with a specific software developed to improve the visual resolution and to facilitate the neuroadaptation (Optictrain, Proconsi SL, León, Spain), and the other 15 patients were included in the placebo group that performed training with a video game not specifically developed for visual training and not containing sinusoidal gratings or specific exercises to activate the circuits of attention, learning, and cognitive control. In the current study, all participants finished the experiment and attended all visits, although compliance with the training was not 100% in all cases (see Section 3.2 and Figure 1).

The Optictrain software was designed with the specific purpose of training contrast sensitivity using Gabor stimuli of different spatial frequencies that are introduced each 1–1.5 min during a driving task game. This game was developed for the study’s purposes, with the objective of combining specific visual stimuli with an attractive task for the subject. The story, scene characteristics, and performance of the game are illustrated in Figure 1. The game consists of driving a car in the first person, with the controls and the road displayed. During the journey, obstacles, such as animals, cones, barriers, and boxes, appear every 2–3 s and have to be avoided by using the arrow keys. In addition, other objects have to be collected by the player by pressing them to get points. The Gabor stimuli were included in the traffic signs that remained static for a period until the player provided a response. Specifically, the subject has to detect the orientation of the fringes of the presented Gabor gratings: vertical, horizontal, 45° inclined to the right, or 45° inclined to the left. For this purpose, four orientations are presented on the screen, and the subject has to mark which of them represent the orientation of the fringes. Not providing a response during this period is counted as a failure.

##### Gabor Stimuli

The Optictrain software uses Gabor patches, which are sinusoidal gratings with a Gaussian envelope that have been previously used in visual training studies (especially in amblyopia) as stimuli [24,25,26]. It should be considered that experimental research has previously demonstrated that gratings with a neutral background can cause selective cortical responses for orientation and contrast, which additionally correlates with functional resonance magnetic imaging findings [33,34]. In our study, the training always began close to the threshold limit measured for each patient with the Optictrain-CS test (basal level). Specifically, the training began with a contrast value that was 10% over the threshold value measured for the spatial frequencies of 0.5, 1.0, 1.5, 3.0, 4.5, and 6.0 cpd. According to the subject’s responses, the contrast of the stimuli was varied following the rules of the Best PEST method [32]. In each session, 5 presentations of each of the evaluated spatial frequencies with their corresponding contrasts were performed. Each of these presentations had a maximum duration of 5 s. If the subject detected contrasts below the threshold during the training, these new values were used as the threshold of reference for the next session to be performed with the game. Otherwise, the next session started with the last threshold used. The same number of presentations of Gabor stimuli for the same spatial frequencies was used in all sessions.

##### Placebo Software

The placebo software was of a free game from PlayStore (Fun Kid Racing 3.53 for Android) based on a driving game experience in which the subject must move right or left to avoid crashes with other vehicles and obstacles on the road. The two pieces of software have similar game execution and trainee goals, with the main difference being the specific visual stimulation by Gabor stimuli of the study group during the driving experience with Optictrain software. 

##### Installation an Indications

Each subject remotely performed the visual training program at home with a previously colorimetrically characterized handheld device model, the Samsung Galaxy Tab A (Samsung, Suwon, South Korea), in which the Optictrain or the placebo software was previously installed. Ten tablets were acquired and used for the training, and consequently only ten subjects could be simultaneously recruited for the trial. Subjects were trained in the use of the software, encouraging them to keep the visualization distance constant at 40 cm. Specifically, a specific position of the arms that allowed for 40 cm of distance between the eyes and the screen was defined for each participant.

#### 2.3.3. Compliance

Subjects were instructed to perform the training task (both Optictrain and placebo) 30 min per day for 20 consecutive days (600 min of training in total). Compliance was checked at the last visit in both groups in the compliance section of the Optictrain software in the study group and with an app usage application (previously downloaded in the placebo tablets) in the placebo group. Participants were informed about this monitorization (included in the informed consent) and gave approval for this issue before participating in this study. Furthermore, participants were not contacted during the training to encourage its participation because we also wanted to test the level of engagement with the software.

#### 2.3.4. Statistical Analysis

The software SPSS v. 22.0 for Windows (IBM, Armonk, NY, USA) was used to perform the statistical analysis of the obtained data. First, a Kolmogorov–Smirnov test was used to confirm whether the data samples were normally distributed. Student’s *t*-test for unpaired data or a Mann–Whitney test was used to analyze the significance of differences between groups depending on whether the normality condition could be assumed. All statistical tests were 2-tailed, and *p*-values of less than 0.05 were considered statistically significant.

The independent variables of the current clinical trial were the following: spatial frequency, contrast of the presented stimuli, type of trifocal intraocular lens, and viewing distance. The following dependent variables were defined: compliance with the visual training, visual acuity, manifest refraction (sphere, cylinder, and spherical equivalent), contrast sensitivity, and defocus curve. Uncorrected monocular and binocular visual acuities were measured at far (4 m), intermediate (1 m), and near (40 cm) distances. Contrast sensitivity was only measured at far and near distances. All contrast sensitivity measurements obtained in the current study were transformed to the decimal logarithmic scale.

## 3. Results

### 3.1. General Description of the Samples and Groups

A total of 30 subjects (23 women and 7 men) were enrolled in this study: 15 subjects were in the placebo group (12 women and 3 men) and 15 were in the study group (11 women and 4 men). The mean patient age was 63.9 ± 6.6 years (range: 50–73 years) in the placebo group and 60.6 ± 6.1 years (range: 47–70 years) in the study group, with no statistically significant differences between them (*p* = 0.22). The visual acuity characteristics of both groups at the baseline visit (before the training) are summarized in Table 1. Mean axial length values for right and left eyes were, respectively, 22.95 ± 0.99 mm (range: 21.30–23.75 mm) and 23.77 ± 0.69 mm (range: 22.91–24.94 mm) in the placebo group, and they were, respectively, 23.34 ± 0.64 mm (range: 22.27–24.22 mm) and 23.44 ± 1.23 mm (range: 21.23–24.76 mm) in the study group, with no significant differences between them (*p* = 0.17 and 0.71, respectively). Concerning the IOL power implanted, the mean values for right and left eyes were, respectively, 22.71 ± 2.44 D (range: 18.00–27.50 D) and 22.75 ± 2.47 D (range: 18.50–28.00 D) in the placebo group, and they were, respectively, 20.57 ± 3.92 D (range: 12.50–26.00 D) and 21.04 ± 3.89 D (range: 12.50–26.00 D) in the study group, with no statistically significant differences between groups (*p* = 0.16 and 0.31, respectively). Likewise, no statistically significant differences between placebo and study groups were found at baseline in photopic pupil size for right (2.78 ± 0.69 vs. 2.86 ± 0.48 mm; *p* = 0.99) and left eyes (3.10 ± 0.58 vs. 2.98 ± 0.51 mm; *p* = 0.68) as well as in scotopic pupil size for right (4.50 ± 0.71 vs. 4.28 ± 0.74 mm; *p* = 0.37) and left eyes (4.43 ± 0.52 vs. 4.32 ± 0.81 mm; *p* = 0.35).

### 3.2. Compliance

**Figure 2:**
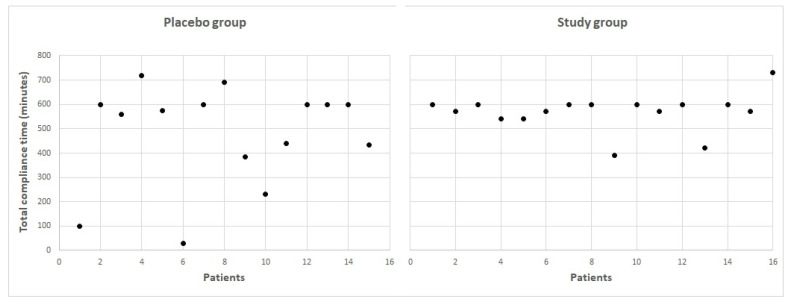
Total compliance time for all the patients included in the two evaluated sample groups.

The mean visual training compliance time in minutes was 468.9 ± 214.0 (range: 30–720 min) in the placebo group and 555.0 ± 67.3 (range: 390–600 min) in the study group, with no statistically significant differences between groups (*p* = 0.64). Figure 2 shows the distribution of the compliance time of the two groups of subjects evaluated in the current study. As is shown, two patients in the placebo group had limited compliance due to the low engagement with the game according to patient feedback. However, no significant correlation was found between the compliance time and the changes achieved in visual acuity and contrast sensitivity parameters in the placebo group (−0.322 ≤ r ≤ 0.181, *p* ≥ 0.263) and the study group (−0.227 ≤ r≤ 0.471, *p* ≥ 0.104).

### 3.3. Refractive Changes

The mean spherical equivalents in right and left eyes, respectively, before the training were 0.04 ± 0.32 D (range: from −0.50 to +1.00 D) and 0.27 ± 0.42 D (range: from 0.00 to +1.25 D) in the placebo group, and they were, respectively, 0.00 ± 0.39 D (range: from −0.75 to +0.50 D) and 0.20 ± 0.49 D (range: from −0.50 to +1.25 D) in the study group, with no statistically significant differences between groups (*p* = 0.83 and 0.48, respectively). After finishing the visual training, mean spherical equivalents in right and left eyes, respectively, after the training were 0.09 ± 0.31 D (range: from −0.50 to +0.62 D) and 0.02 ± 0.35 D (range: from −0.50 to +0.50 D) in the placebo group, and they were, respectively, −0.07 ± 0.34 D (range: from −0.75 to +0.50 D) and 0.03 ± 0.44 D (range: from −0.88 to +1.00 D) in the study group, with no statistically significant differences between groups (*p* = 0.26 and 0.94, respectively).

### 3.4. Visual Acuity Changes

**Figure 3:**
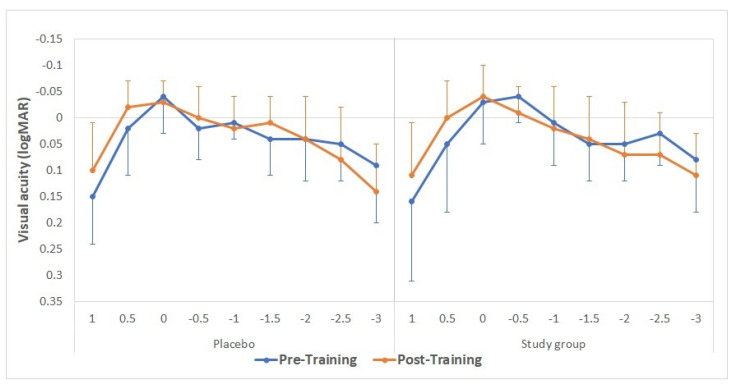
Mean defocus curves obtained before (continuous lines) and after (discontinuous lines) the training, considering the defocus levels from −3.00 to +1.50 D (with a 0.50 D step size) for the placebo (green) and study groups (purple).

Table 1 and Table 2 summarize the monocular and binocular visual acuity data, respectively, obtained before and after training. The tables show no statistically significant differences between placebo and study groups were obtained in any evaluated visual acuity parameter (*p* ≥ 0.15). The mean defocus curves obtained before and after the training in the study and placebo groups are displayed in Figure 3. No statistically significant differences were found between groups in the visual acuity measured for any vergence demand before the training (*p* > 0.56 in all cases), except for the defocus level of −0.50 D (*p* = 0.02). After the training, no statistically significant differences were found between groups for any vergence (*p* > 0.27 in all cases).

### 3.5. Contrast Sensitivity Changes

At baseline, the mean near (40 cm) logCS obtained with the Pelli–Robson test was 1.69 ± 0.09 (from 1.65 to 1.95 logCS) and 1.78 ± 0.13 (from 1.65 to 1.95 logCS) in the placebo and study groups, respectively. This difference was close to the limit considered for reaching statistical significance (*p* = 0.051). After the training, the mean near logCS was 1.68 ± 0.10 (from 1.50 to 1.95 logCS) and 1.77 ± 0.12 (from 1.65 to 1.95 logCS) in the placebo and study groups, respectively, thus reaching a statistically significant difference (*p* = 0.047).

**Figure 4:**
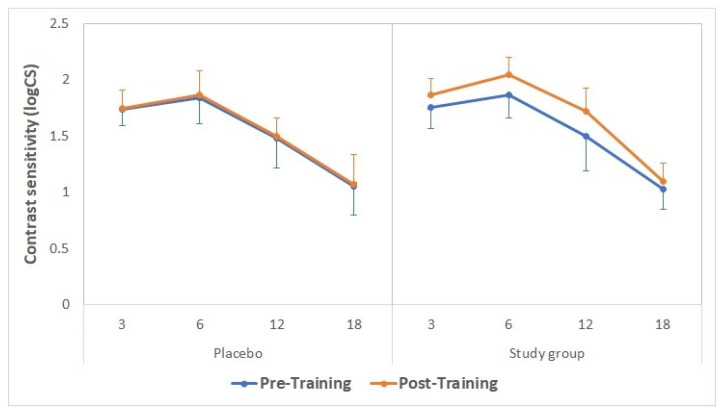
Distance contrast sensitivity values obtained with the CSV1000 test before (continuous lines) and after (discontinuous lines) the training in the placebo (green) and study groups (purple).

Distance (4 m) contrast sensitivity data obtained with the CSV1000 test before and after the training are summarized in Figure 4. No statistically significant differences were found between groups in contrast sensitivity for any spatial frequency before the training (*p* ≥ 0.74). After the training, statistically significant differences were found between groups for the spatial frequencies of 6 and 12 cpd (*p* = 0.02 and 0.01, respectively), thus showing the best outcome in the study group.

The near contrast sensitivity data measured with the Optopad test before and after the training are summarized in Table 3. No statistically significant differences were found between groups for any frequency before the training (*p* ≥ 0.51). After the training, the values of contrast sensitivity obtained in the study group for the spatial frequencies of 1.5, 3, and 6 cpd were higher for most of subjects than those obtained in the placebo group, but differences between groups did not reach statistical significance (*p* ≥ 0.08). When separately analyzing changes in contrast sensitivity measured with the Optopad test in each group, a statistically significant improvement was found in the contrast sensitivity value corresponding to the spatial frequency of 1.5 (*p* = 0.026) in the study group, whereas no significant changes were detected in the contrast sensitivity measured for any of the spatial frequencies evaluated in the placebo group (*p* ≥ 0.283). 

The near contrast sensitivity data measured with the Optictrain-CS test are summarized in Table 4. As is shown, no statistically significant differences between groups were found before the training in terms of contrast sensitivity values for any of the evaluated spatial frequencies (*p* ≥ 0.13). After the training, significantly better values of near contrast sensitivity were only found in the study group compared to the placebo group for the spatial frequency of 1.5 cpd (*p* = 0.02). 

### 3.6. Evaluation of the Effect for Both Types of IOLs

In addition to this analysis, an additional comparative evaluation was performed between placebo and study groups in the two subgroups of patients implanted with the two types of trifocal diffractive IOL, Finevision (placebo 6 vs. study 7) and RayOne (placebo 9 vs. study 8). A trend towards better contrast sensitivity values was found in both study subgroups (the Finevision and RayOne study subgroups) compared to the placebo subgroups. Specifically, no significant differences were found in any visual and contrast sensitivity parameters between the Finevision study and placebo subgroups after training (*p* ≥ 0.078). However, a difference in the limit of statistical significance (*p* = 0.055) was found between the RayOne study and placebo subgroups after training in near (40 cm) contrast sensitivity for 3 cpd measured with the Optictrain-CS test.

### 3.7. Data Re-Analysis with Those Cases with Compliance Time of 300 Min or More

A re-analysis was conducted for those participants with a compliance time of 300 min or more (12 patients in the placebo group and 15 patients in the study group). No significant differences were found between groups in binocular distance (*p* = 0.936), intermediate (*p* = 0.687), and near visual acuity (*p* = 0.609). Concerning distance (4 m) contrast sensitivity, significantly better values were found in the study group compared to the placebo group for the spatial frequencies of 6 (1.86 ± 0.23 vs. 2.05 ± 0.15, respectively; *p* = 0.029) and 12 cycles/degree (1.48 ± 0.16 vs. 1.72 ± 0.21, respectively; *p* = 0.009). In contrast, as in the analysis of the whole sample, no statistically significant differences were found between groups in near contrast sensitivity values for any of the evaluated spatial frequencies (*p* ≥ 0.107). 

Regarding the significant longitudinal changes in contrast sensitivity values in each group, only a significant decrease in near contrast sensitivity measured with the Optictrain-CS test was found for the spatial frequency of 4.5 cycle/degree (pre-training 2.14 ± 0.20 vs. post-training 1.82 ± 0.23; *p* = 0.009). In contrast, in the study group, significant increases in distance contrast sensitivity were found for the spatial frequencies of 6 (pre-training 1.87 ± 0.21 vs. post-training 2.05 ± 0.15; *p* = 0.014) and 12 cycle/degree (pre-training 1.50 ± 0.31 vs. post-training 1.72 ± 0.21; *p* = 0.016), as well as in near contrast sensitivity measured with the Optopad-CSF test for the spatial frequencies of 1.5 (pre-training 3.46 ± 0.09 vs. post-training 3.62 ± 0.24; *p* = 0.026) and 3.0 cycles/degree (pre-training 3.74 ± 0.14 vs. post-training 3.92 ± 0.27; *p* = 0.048).

## 4. Discussion

Concerning visual acuity data at 4 m, 1 m, and 40 cm, no significant differences were found between patients from the placebo and study groups, which confirms that the impact of the visual training program was minimal. It should be considered that the visual acuity outcomes obtained with the two types of trifocal IOLs included in the current series were already good in the initial postoperative period, as has been reported in several previous investigations [35,36,37]. Tan and Fong [25] evaluated the efficacy and safety of a Gabor gratings-based visual training to improve visual acuity and contrast sensitivity in low myopic eyes, and they obtained a significant improvement in uncorrected visual acuity. However, in the current trial, the residual refraction was minimal because the high level of predictability achieved with the refractive correction of the IOL made the adaptation to a specific level of residual refraction unnecessary. 

The superiority of the impact on contrast sensitivity at 4 m of the use of the visual training program evaluated over the placebo was evident, with significantly better post-training contrast sensitivity values for the spatial frequencies of 6 and 12 cpd in the study group. This result is consistent with the outcome obtained in the clinical study of Kaymak et al. [15]. In this study, these authors trained one eye of 16 participants that had undergone bilateral cataract surgery with the implantation of multifocal IOLs in six sessions over 2 weeks, with the untrained fellow eye serving as the control. The training was conducted using a computerized system based on the concept of the perceptual learning of discrimination line orientations, with training sessions of a mean duration of 30 ± 5 min. These authors found significant improvements in orientation visual acuity and contrast sensitivity compared to control eyes. Specifically, these authors evaluated the area under the contrast sensitivity curve under photopic, mesopic, and mesopic with glare conditions, obtaining significant changes after visual training in all of them. Likewise, Tan and Fong [25] reported significant improvements in uncorrected contrast sensitivity for low, medium, and high spatial frequencies measured using a wall-mounted sine-wave contrast test (SWCT) chart (Stereo Optical Co., Chicago, IL, USA) at 3 m in low myopic eyes trained with Gabor gratings. Caution should be taken when comparing these previous investigations that evaluated the impact of Gabor grating-based visual trainings with the outcomes of the current trial, as significant differences among studies are present in terms of illumination conditions, the pupil characteristics of the enrolled patients, the level of the gamification of the visual training, the psychophysical method used for stimulating and evaluating the contrast sensitivity, and the resolution and calibration of the screen in which the stimuli were presented.

Concerning near contrast sensitivity (40 cm), three different tests were used to investigate the potential benefit of the evaluated visual training program: Pelli–Robson (based on the use of letters and one main spatial frequency), Optopad (several spatial frequencies and sinusoidal gratings), and Optictrain-CS (several spatial frequencies and the use of Gabor patches). With the Optopad and Optictrain-CS tests, better values of near contrast sensitivity were measured for low and medium spatial frequencies in the study group, although only the difference between study and placebo groups for the spatial frequency of 1.5 cpd measured with the Optictrain-CS test reached statistical significance. With the Pelli–Robson test, the significant difference found among groups postoperatively should be considered with care because, preoperatively, there was a difference close to the limit of statistical significance among groups in the measures obtained with this test. Kaymak et al. [15] reported significant improvements in low-contrast (12.5%) near visual acuity in a group of eyes implanted with multifocal IOLs and trained with the sinusoidal grating for 6 weeks. 

It should be considered that high spatial frequencies represent abrupt spatial changes in an image, and their detection corresponds to the detection of fine details of the configuration [38]. Therefore, there is a relationship between visual acuity and contrast sensitivity threshold for high spatial frequencies [39]. In our trial, no significant improvement in visual acuity or contrast sensitivity for high spatial frequencies were found, which seems to be consistent with this relationship among these two parameters. On the other hand, low spatial frequencies represent global information about shape, such as general orientation and proportions [39]. This suggests an improvement in the ability to detect and process local and global aspects of real-world scenes, as well as a potential better control of the selection of the focused image generated by the implant on the retina. In any case, the effect of selective changes of the global function of contrast sensitivity with frequency should be analyzed with caution in a real-world space, as it will depend on the frequency content of the original image [40]. Luque et al. [41] simulated global and selective losses of achromatic contrast sensitivity in low (1 cpd), medium (4 cpd), and high frequency (16 cpd) areas using colored images. As overall losses in achromatic sensitivity increased, the scene became quasi-isoluminant, causing image segmentation and pattern recognition tasks to become increasingly difficult. These authors showed the effects of losses in three different frequency ranges (1, 4, and 16 cpd) that may correspond with the loss of a set of specific frequency-selective sensors (due the bandpass characteristics of the striate cortex cell-mediating pattern detection). Losses in the high frequency range result in defocus, whereas losses in the low and medium frequency ranges create haloes in an image (dark and bright bands around luminance contours, respectively). This should be further investigated to understand the meaning of this improvement in terms of daily activities.

This clinical trial had some limitations that should be acknowledged. First, the sample size was limited, though some significant changes could still be detected. As this clinical trial is still ongoing and more patients are being recruited and evaluated, future analysis of the real efficacy of this visual training program, including a follow-up in the long term, will be provided. The inclusion of two different types of multifocal IOLs may be considered another limitation, but it should be considered that the two types of trifocal IOLs have a diffractive profile. In any case, the sample was subdivided according to the implanted IOL, and a comparison between study and placebo subgroups was performed for each type of IOL: Finevision (placebo 6 vs. study 7) and RayOne (placebo 9 vs. study 8). For eyes implanted with the Finevision IOL, this analysis showed no significant differences in any visual and contrast sensitivity parameters between study and placebo subgroups after training. However, a difference close to the limit of statistical significance was found between RayOne study and placebo subgroups after training in near contrast sensitivity for 3 cpd (Optictrain-CS). It is difficult to extract consistent conclusions regarding the potential different response of the visual training as a function of the implanted multifocal IOL due to the limitation of the subgroups (all subgroups included 9 or less subjects). Future analyses with larger samples that evaluate the impact of the visual training program on eyes implanted with different types of multifocal IOLs should be performed. In addition, future studies are also needed to investigate the impact of photic phenomena on perception, as well as patient satisfaction with this type of visual training. 

Another potential limitation of the current trial was the control of the distance of visualization during the training. Though subjects were trained to keep a constant distance of 40 cm during the training, it was not monitored with an eye-tracking system. Future studies that combine this training software with an eye tracker to show the potential impact of this issue should be conducted. Finally, this trial only evaluated the immediate impact of the visual training program on the patient’s visual function; the maintenance of the improvement achieved in the medium and long term must be also investigated. In their clinical study with 16 patients implanted with multifocal IOLs, Kaymak et al. [15] confirmed that the effect of the visual training program based on the concept of perceptual learning of discrimination line orientations applied was sustained over a 6 month period.

## 5. Conclusions

In conclusion, the results of this preliminary study suggest that a 3 week visual training program based on the use of Gabor patches in the immediate postoperative period after the bilateral implantation of trifocal diffractive IOLs may be beneficial for improving the visual performance achieved with the implant in both distance and near. These preliminary outcomes should be confirmed in future studies with larger sample sizes and long-term follow-up.

## Figures and Tables

**Table 1:** Monocular VA characteristics of both groups before the training. Mean ± standard deviation (minimum and maximum) values for distance, intermediate, and near vision are represented by groups for right and left eyes. Differences between groups were assessed by a Mann–Whitney test, and statistically significant differences (*p* < 0.05) are marked with an asterisk (*). Distance, intermediate, and near vision were assessed at 4 m, 1 m, and 40 cm, respectively.

			Placebo Group	Study Group	*p*-Value
LogMAR Uncorrected Visual Acuity	Distance (4 m)	Right	0.03 ± 0.09 (−0.08–0.30)	0.05 ± 0.07 (−0.08–0.15)	0.21
		Left	0.07 ± 0.12 (−0.08–0.40)	0.05 ± 0.10 (−0.08–0.22)	0.51
	Intermediate (1 m)	Right	0.04 ± 0.05 (0.00–0.15)	0.10 ± 0.13 (0.00–0.40)	0.35
		Left	0.06 ± 0.10 (0.00–0.30)	0.11 ± 0.12 (0.00–0.40)	0.17
	Near (40 cm)	Right	0.10 ± 0.10 (0.00–0.30)	0.10 ± 0.11 (0.00–0.30)	0.88
		Left	0.15 ± 0.13 (0.00–0.40)	0.09 ± 0.11 (0.00–0.30)	0.24
LogMAR Distance-Corrected Visual Acuity	Distance (4 m)	Right	−0.01 ± 0.04 (−0.08–0.02)	−0.01 ± 0.03 (−0.08–0.05)	0.86
		Left	−0.01 ± 0.05 (−0.08–0.10)	−0.00 ± 0.04 (−0.08–0.10)	0.82
	Intermediate (1 m)	Right	0.05 ± 0.05 (0.00–0.15)	0.09 ± 0.13 (0.00–0.40)	0.65
		Left	0.05 ± 0.07 (0.00–0.22)	0.13 ± 0.15 (0.00–0.54)	0.15
	Near (40 cm)	Right	0.08 ± 0.09 (0.00–0.30)	0.08 ± 0.08 (0.00–0.30)	0.68
		Left	0.09 ± 0.09 (0.00–0.22)	0.09 ± 0.12 (0.00–0.30)	0.83

**Table 2:** Binocular VA characteristics of both groups before the training. Mean ± standard deviation (minimum and maximum) values for distance, intermediate, and near vision are represented by groups. Differences between groups were assessed by the Mann–Whitney test, and statistically significant differences (*p* < 0.05) are marked with an asterisk (*). Distance, intermediate, and near vision were assessed at 4 m, 1 m, and 40 cm, respectively.

		Placebo Group	Study Group	*p*-Value
Before Training	Distance (4 m)	−0.01 ± 0.04 (−0.08–0.05)	−0.01 ± 0.07 (−0.08–0.10)	0.64
	Intermediate (1 m)	0.00 ± 0.03 (−0.08–0.05)	0.03 ± 0.08 (−0.08–0.15)	0.25
	Near (40 cm)	0.05 ± 0.09 (0.00–0.30)	0.05 ± 0.08 (0.00–0.22)	0.92
After Training	Distance (4 m)	−0.04 ± 0.05 (−0.11–0.05)	−0.03 ± 0.06 (−0.08–0.10)	0.76
	Intermediate (1 m)	0.01 ± 0.06 (−0.08–0.15)	0.01 ± 0.08 (−0.08–0.15)	0.67
	Near (40 cm)	0.08 ± 0.12 (0.00–0.30)	0.06 ± 0.08 (0.00–0.22)	0.83

**Table 3:** Near contrast sensitivity results obtained before and after the training with the Optopad test. Statistically significant differences (*p* < 0.05) are marked with an asterisk (*).

		Optopad Contrast Sensitivity Test		
		Placebo Group	Study Group	*p*-Value
Before Training	1.5 cpd	3.45 ± 0.11 (3.30–3.65)	3.46 ± 0.09 (3.37–3.58)	0.80
	3.0 cpd	3.71 ± 0.19 (3.38–4.11)	3.74 ± 0.13 (3.50–3.99)	0.51
	6.0 cpd	3.45 ± 0.24 (3.05–3.86)	3.47 ± 0.19 (3.26–3.86)	0.81
	12.0 cpd	2.94 ± 0.29 (2.53–3.40)	2.97 ± 0.23 (2.53–3.32)	0.77
	24.0 cpd	2.21 ± 0.31 (1.76–2.78)	2.13 ± 0.17 (1.76–2.51)	0.57
After Training	1.5 cpd	3.47 ± 0.16 (3.05–3.72)	3.62 ± 0.24 (3.37–4.13)	0.13
	3.0 cpd	3.76 ± 0.20 (3.50–4.11)	3.92 ± 0.27 (3.38–4.23)	0.08
	6.0 cpd	3.44 ± 0.28 (3.05–3.99)	3.53 ± 0.25 (3.05–3.99)	0.27
	12.0 cpd	2.99 ± 0.55 (1.64–3.82)	2.88 ± 0.34 (2.27–3.24)	0.33
	24.0 cpd	2.11 ± 0.23 (1.76–2.51)	2.13 ± 0.19 (1.76–2.37)	0.85

**Table 4:** Near contrast sensitivity results obtained before and after the training with the Optictrain-CS test. Mean ± standard deviation (minimum and maximum) of contrast sensitivity values are represented in logarithmic units (logCS). Differences between groups were assessed with the Mann–Whitney test, and statistically significant differences (*p* < 0.05) are marked with an asterisk (*).

		Optictrain-CS Contrast Sensitivity Test		
		Placebo Group	Study Group	*p*-Value
Before Training	0.5 cpd	1.88 ± 0.24 (1.54–2.30)	2.02 ± 0.20 (1.78–2.30)	0.13
	1.0 cpd	2.22 ± 0.14 (2.01–2.30)	2.17 ± 0.19 (1.78–2.30)	0.54
	1.5 cpd	2.20 ± 0.15 (2.01–2.30)	2.19 ± 0.17 (1.85–2.30)	0.89
	3.0 cpd	2.10 ± 0.19 (1.78–2.30)	2.10 ± 0.23 (1.66–2.30)	0.86
	4.5 cpd	2.10 ± 0.29 (1.32–2.30)	1.99 ± 0.28 (1.32–2.30)	0.18
	6.0 cpd	1.92 ± 0.32 (1.19–2.30)	1.95 ± 0.31 (1.19–2.30)	0.78
After Training	0.5 cpd	1.98 ± 0.22 (1.52–2.30)	1.96 ± 0.31 (1.51–2.30)	0.94
	1.0 cpd	2.15 ± 0.23 (1.58–2.30)	2.11 ± 0.18 (1.78–2.30)	0.39
	1.5 cpd	2.13 ± 0.15 (2.01–2.30)	2.26 ± 0.11 (2.01–2.30)	0.02 *
	3.0 cpd	2.05 ± 0.20 (1.58–2.30)	2.18 ± 0.30 (1.58–2.91)	0.20
	4.5 cpd	1.85 ± 0.25 (1.54–2.30)	1.94 ± 0.28 (1.58–2.30)	0.35
	6.0 cpd	1.76 ± 0.24 (1.52–2.30)	1.86 ± 0.30 (1.40–2.30)	0.32

## Data Availability

Data are available on request due to privacy/ethical restrictions.

## References

[1] Khandelwal S.S., Jun J.J., Mak S., Booth M.S., Shekelle P.G. (2019). Effectiveness of multifocal and monofocal intraocular lenses for cataract surgery and lens replacement: A systematic review and meta-analysis. Graefes Arch. Clin. Exp. Ophthalmol..

[2] Palomino-Bautista C., Sánchez-Jean R., Carmona-Gonzalez D., Piñero D.P., Molina-Martín A. (2021). Depth of field measures in pseudophakic eyes implanted with different type of presbyopia-correcting IOLs. Sci. Rep..

[3] Palomino-Bautista C., Sánchez-Jean R., Carmona-González D., Piñero D.P., Molina-Martín A. (2020). Subjective and objective depth of field measures in pseudophakic eyes: Comparison between extended depth of focus, trifocal and bifocal intraocular lenses. Int. Ophthalmol..

[4] Zamora-de La Cruz D., Zúñiga-Posselt K., Bartlett J., Gutierrez M., Abariga S.A. (2020). Trifocal intraocular lenses versus bifocal intraocular lenses after cataract extraction among participants with presbyopia. Cochrane Database Syst. Rev..

[5] Modi S., Lehmann R., Maxwell A., Solomon K., Cionni R., Thompson V., Horn J., Caplan M., Fisher B., Hu J.G. (2021). Visual and patient-reported outcomes of a diffractive trifocal intraocular lens compared with those of a monofocal intraocular lens. Ophthalmology.

[6] Plaza-Puche A.B., Alio J.L., Sala E., Mojzis P. (2016). Impact of low mesopic contrast sensitivity outcomes in different types of modern multifocal intraocular lenses. Eur. J. Ophthalmol..

[7] Fernández J., Rodríguez-Vallejo M., Martínez J., Burguera N., Piñero D.P. (2021). What we have learnt from 30 years living with positive dysphotopsia after intraocular lens implantation?: A review. Exp. Rev. Ophthalmol..

[8] Seiler T.G., Wegner A., Senfft T., Seiler T. (2019). Dissatisfaction after trifocal IOL implantation and its improvement by selective wavefront-guided LASIK. J. Refract. Surg..

[9] Kamiya K., Hayashi K., Shimizu K., Negishi K., Sato M., Bissen-Miyajima H., Survey Working Group of the Japanese Society of Cataract and Refractive Surgery (2014). Multifocal intraocular lens explantation: A case series of 50 eyes. Am. J. Ophthalmol..

[10] Gibbons A., Ali T.K., Waren D.P., Donaldson K.E. (2016). Causes and correction of dissatisfaction after implantation of presbyopia-correcting intraocular lenses. Clin. Ophthalmol..

[11] Woodward M.A., Randleman J.B., Doyle Stulting R. (2009). Dissatisfaction after multifocal intraocular lens implantation. J. Cataract. Refract. Surg..

[12] Coco-Martin M.B., Valenzuela P.L., Maldonado-López M.J., Santos-Lozano A., Molina-Martín A., Piñero D.P. (2019). Potential of video games for the promotion of neuroadaptation to multifocal intraocular lenses: A narrative review. Int. J. Ophthalmol..

[13] Rosa A.M., Miranda A.C., Patricio M., McAlinden C., Silva F.L., Murta J.N., Castelo-Branco M. (2017). Functional magnetic resonance imaging to assess the neurobehavioral impact of dysphotopsia with multifocal intraocular lenses. Ophthalmology.

[14] Rosa A.M., Miranda Â.C., Patrício M.M., McAlinden C., Silva F.L., Castelo-Branco M., Murta J.N. (2017). Functional magnetic resonance imaging to assess neuroadaptation to multifocal intraocular lenses. J. Cataract. Refract. Surg..

[15] Kaymak H., Fahle M., Ott G., Mester U. (2008). Intraindividual comparison of the effect of training on visual performance with ReSTOR and Tecnis diffractive multifocal IOLs. J. Refract. Surg..

[16] Mester U., Fahle M., Ott G., Kaymak H. (2008). Functional vision training after MIOL implantation. Ophthalmologe.

[17] Hernández-Rodríguez C.J., Fukumitsu H., Ruiz-Fortes P., Soto-Negro R., Merino-Suárez M., Piñero D.P. (2021). Efficacy of perceptual learning-based vision training as an adjuvant to occlusion therapy in the management of amblyopia: A pilot study. Vision.

[18] Coco-Martin M.B., Piñero D.P., Leal-Vega L., Hernández-Rodríguez C.J., Adiego J., Molina-Martín A., de Fez D., Arenillas J.F. (2020). The potential of virtual reality for inducing neuroplasticity in children with amblyopia. J. Ophthalmol..

[19] Halicka J., Bittsansky M., Sivak S., Piñero D.P., Ziak P. (2021). Virtual reality visual training in an adult patient with anisometropic amblyopia: Visual and functional magnetic resonance outcomes. Vision.

[20] Liu X.Y., Zhang J.Y. (2018). Dichoptic training in adults with amblyopia: Additional stereoacuity gains over monocular training. Vis. Res..

[21] Žiak P., Holm A., Halička J., Mojžiš P., Piñero D.P. (2017). Amblyopia treatment of adults with dichoptic training using the virtual reality oculus rift head mounted display: Preliminary results. BMC Ophthalmol..

[22] Vedamurthy I., Nahum M., Bavelier D., Levi D.M. (2015). Mechanisms of recovery of visual function in adult amblyopia through a tailored action video game. Sci. Rep..

[23] Hess R.F., Babu R.J., Clavagnier S., Black J., Bobier W., Thompson B. (2014). The iPod binocular home-based treatment for amblyopia in adults: Efficacy and compliance. Clin. Exp. Optom..

[24] Yalcin E., Balci O. (2014). Efficacy of perceptual vision therapy in enhancing visual acuity and contrast sensitivity function in adult hypermetropic anisometropic amblyopia. Clin. Ophthalmol..

[25] Tan D.T., Fong A. (2008). Efficacy of neural vision therapy to enhance contrast sensitivity function and visual acuity in low myopia. J. Cataract. Refract. Surg..

[26] Hernández-Rodríguez C.J., Piñero D.P., Molina-Martín A., Morales-Quezada L., de Fez D., Leal-Vega L., Arenillas J.F., Coco-Martín M.B. (2020). Stimuli characteristics and psychophysical requirements for visual training in amblyopia: A narrative review. J. Clin. Med..

[27] Ten Brinke L.F., Davis J.C., Barha C.K., Liu-Ambrose T. (2017). Effects of computerized cognitive training on neuroimaging outcomes in older adults: A systematic review. BMC Geriatr..

[28] Palaus M., Marron E.M., Viejo-Sobera R., Redolar-Ripoll D. (2017). Neural basis of video gaming: A systematic review. Front. Hum. Neurosci..

[29] McAnanya J.J., Alexander K.R. (2006). Contrast sensitivity for letter optotypes vs. gratings under conditions biased toward parvocellular and magnocellular pathways. Vis. Res..

[30] de Fez D., Luque M.J., García-Domene M.C., Camps V., Piñero D. (2016). Colorimetric characterization of mobile devices for vision applications. Optom. Vis. Sci..

[31] Vrhel M.J., Trussell H.J. (1999). Color device calibration: A mathematical formulation. IEEE. Trans. Image Process..

[32] Lieberman H.R., Pentland A.P. (1982). Microcomputer-based estimation of psychophysical thresholds: The Best PEST. Behav. Res. Method. Instrum..

[33] Tootell R., Hadjikhani N., Vanduffel W., Liu A.K., Mendola J.D., Sereno M.I., Dale A.M. (1998). Functional analysis of primary visual cortex (V1) in humans. Proc. Natl. Acad. Sci. USA.

[34] Georgeson M. (2004). Visual altereffects: Cortical neurons change their tune. Curr. Biol..

[35] Hienert J., Stjepanek K., Hirnschall N., Ruiss M., Zwickl H., Findl O. (2021). Visual performance of two diffractive trifocal intraocular lenses: A randomized trial. J. Refract. Surg..

[36] Ribeiro F., Ferreira T.B. (2020). Comparison of clinical outcomes of 3 trifocal IOLs. J. Cataract Refract. Surg..

[37] Poyales F., Garzon N. (2019). Comparison of 3-month visual outcomes of a spherical and a toric trifocal intraocular lens. J. Cataract Refract. Surg..

[38] Bar M. (2004). Visual objects in context. Nat. Rev. Neurosci..

[39] Michael R., van Rijn L.J., van den Berg T.J.T.P., Barraquer R.I., Grabner G., Wilhelm H., Coeckelbergh T., Emesz M., Marvan P., Nischler C. (2009). Association of lens opacities, intraocular straylight, contrast sensitivity and visual acuity in European drivers. Acta Ophthalmol..

[40] de Fez M.D., Luque M.J., Viqueira V. (2002). Enhancement of contrast sensitivity and losses of chromatic discrimination with tinted lenses. Optom. Vis. Sci..

[41] Luque M.J., Capilla P., de Fez M.D., García-Domene M.C. (2010). Images perceived after chromatic or achromatic contrast sensitivity losses. Optom. Vis. Sci..

